# Effects of Oral Resveratrol Supplementation on Glycogen Replenishment and Mitochondria Biogenesis in Exercised Human Skeletal Muscle

**DOI:** 10.3390/nu12123721

**Published:** 2020-12-02

**Authors:** Chun-Ching Huang, Chia-Chen Liu, Jung-Piao Tsao, Chin-Lin Hsu, I-Shiung Cheng

**Affiliations:** 1 Department of Exercise and Health Sciences, National Taipei University of Nursing and Health Science, Taipei City 112, Taiwan; cchuang@ntunhs.edu.tw; 2 Department of Physical Education, National Taichung University of Education, Taichung City 403, Taiwan; jamesliu@gm.ntcu.edu.tw (C.-C.L.); tjp1984@mail.ntcu.edu.tw (J.-P.T.); 3 Department of Nutrition, Chung Shan Medical University Hospital, Taichung 404, Taiwan; 4 School of Nutrition, Chung Shan Medical University, Taichung 404, Taiwan

**Keywords:** ergogenic aids, cycling exercise, muscle physiology, energy metabolism

## Abstract

The present study aimed to investigate the effect of oral resveratrol supplementation on the key molecular gene expressions involved in mitochondria biogenesis and glycogen resynthesis in human skeletal muscle. Nine young male athletes participated in the single-blind and crossover designed study. All subjects completed a 4-day resveratrol and placebo supplement in a randomized order while performing a single bout of cycling exercise. Immediately after the exercise challenge, the subjects consumed a carbohydrate (CHO) meal (2 g CHO/Kg body mass) with either resveratrol or placebo capsules. Biopsied muscle samples, blood samples and expired gas samples were obtained at 0 h and 3 h after exercise. The muscle samples were measured for gene transcription factor expression by real-time PCR for glucose uptake and mitochondria biogenesis. Plasma glucose, insulin, glycerol, non-esterified fatty acid concentrations and respiratory exchange ratio were analyzed during post-exercise recovery periods. The results showed that the muscle glycogen concentrations were higher at 3 h than at 0 h; however, there were no difference between resveratrol trial and placebo trial. There were no significantly different concentrations in plasma parameters between the two trials. Similarly, no measured gene expressions were significant between the two trials. The evidence concluded that the 4-day oral resveratrol supplementation did not improve post-exercise muscle glycogen resynthesis and related glucose uptake and mitochondrial biosynthesis gene expression in men.

## 1. Introduction

Muscle glycogen is a critical energy source in high-intensity exercise and a key factor related to exercise fatigue [1]. Therefore, it is a crucial strategy in sports nutrition to identify the critical selection of nutritional supplements on rapid recovery muscle glycogen replenishment after high-intensity exercise. The gene expression of GLUT4 is positively influenced by AKT and AS160 (TBC1D4) [2]. The study demonstrated that the amount of musculoskeletal GLUT4 expression is directly proportional to the speed of muscle synthesizing glycogen after exercise [3]. Previous human and rodent studies showed that post-exercise glucose absorption and muscle glycogen replenishment were enhanced by exercise-induced promotion translocation of GLUT4-containing vesicles into the muscle plasma membrane [4,5]. In addition, hexokinase Ⅱ (HKII) is a critical regulatory factor in glucose metabolic turnover into glucose-6-phosphate. The energy status of physical activity clearly affects glucose metabolism, which may take the path of glucose glycolysis or glycogen synthesis [6]. Human experiments involving high-intensity exercise which investigated musculoskeletal glucose uptake-related gene expression of Rab GTPase-TBC1D4/AS1601 showed that TBC1D1/AS160 phosphorylation on multiple sites was activated after a 30-min cycling exercise bout at 70% VO_2 max_ [7] and another human study showed positive phosphorylation of TBC1D1 and TBC1D4 under high-intensity resistance exercise performed with a 60-min bout of one-legged knee extensor exercise at 80% of peak workload [8]. The relevant effects of resveratrol on the glucose uptake genes present in the human musculoskeletal system under high-intensity exercise should be investigated in comparison to rodent studies.

Musculoskeletal mitochondria are the main cell organelles generating energy during exercise. Through oxidative phosphorylation and the electron transport chain, they generate adenosine triphosphate (ATP) as a source of energy for the body [9]. NRF1, together with NRF2, increases the expression of mitochondrial transcription factor A (TFAM), which enhance mitochondrial biosynthesis efficiency [10] and bioenergy ATP yield [11]. The NRF1-NRF2-TFAM axis was upregulated by the activation of SIRT1 and PGC-1α under oxidative stress conditions [12]. A human experiment revealed that polyphenol quercetin supplements substantially increase mitochondrial biosynthesis in SIRT1 and PGC-1α and their gene expression, increasing the running distance of participants [13].

A previous study demonstrated that oral polyphenol supplementation is a critical sports nutrition strategy to facilitate mitochondrial biosynthesis for athletes to delay the onset of exercise fatigue and to enhance performance [13,14]. Resveratrol (RES), as a nutritional supplement, is a natural polyphenol compound accompanied by functions of increasing antioxidant activity [15], anti-inflammation [16], anti-aging [17] and diabetes prevention [18]. With four-week RES supplementation (receiving oral 2 × 5 mg resveratrol/day), patients with type 2 diabetes exhibited a substantial reduction in oxidative stress and improved insulin resistance via the Akt pathway [19]. The review article mentioned the potential of the resveratrol estradiol effect as a therapy for human joint disorders, an effect including AMPK and PGC-1 induction [20]. Skeletal muscle measurement during human experiments involving 12-week oral resveratrol (3 g per day) supplementation included changes in AMPK, phosphorylated-AMPK-Thr172 (p-AMPK) and GLUT4 expression levels. The significant finding of increased SIRT1 and AMPK expression was noted in a resveratrol trial [21]. Eight young males performed endurance cycling training at 70% VO_2max_ for 30 min/day for four weeks. Endurance training elicits profound adaptations of skeletal muscle, including increased expression of AMPK, which in turn enhances GLUT4 level [22]. The above-mentioned human studies show that an agonist of the estradiol receptor, a critical component in RES, substantially increased after RES supplementation and improve whole-body insulin sensitivity. In our study, we are interested in the influence of RES on the upregulation of GLUT4 gene expression and the increase of the absorption efficiency of glucose in human muscle tissue.

The ERR-α, an estrogen-related receptor, activates PGC-1α and promotes the performance of various signal enzymes in mitochondria biosynthesis. In addition, the results from an animal study providing RES supplements for a short period showed that RES supplementation upregulated NRF-1 expression via SIRT1 and the activity of PGC-1α was increased to promote the effectiveness of mitochondria biosynthesis [21,23]. According to the above-mentioned studies supporting the effect of resveratrol supplementation, the purpose of the present study was to demonstrate the ergogenic effects of 4 consecutive days of oral resveratrol administration on muscle glycogen replenishment and mitochondria biosynthesis after performing 60 min of cycling exercise at 70% VO_2max_.

## 2. Materials and Methods

### 2.1. Research Participants

The research participants consisted of nine healthy young male volunteers (with a mean age of 20.2 ± 0.4 years, a mean height of 174.4 ± 1.1 cm, a mean BMI of 23.1 ± 0.5 kg/m^2^ and a mean maximum oxygen uptake of 51.4 ± 1.7 mL/kg/min). The investigations were carried out following the rules of the Declaration of Helsinki of 1975 and this study was approved on 1 August 2017 by the Institutional Review Board of the University of Taipei, Taipei, Taiwan (approval license IRB-2016-053). All participants joined the experiment voluntarily, for 2 months prior to the formal experiment, and avoided taking supplements with anti-inflammatory agents. All subjects were fully informed of the risks and discomfort associated with the study, and all provided written consent before participation.

### 2.2. Experimental Procedure

Seven days before the exercise experiment, the maximum oxygen consumption (VO_2max_) of the participants was measured. We adopted a single-blind crossover design. All the participants randomly received RES (480 mg/day, a total of 1920 mg) or a placebo for four consecutive days. The two experiments were 14 days apart. Three days before the experiment, all of the participants were provided with oral RES or placebo capsules, and they were under a controlled diet. On the day of the experiment, the participants fasted the previous night, and at 7 a.m., they reported to the laboratory for their weight to be measured and to receive a blood sampling. After a 5-min warm-up, the participants performed 70% VO_2max_ bicycle exercise for 60 min. The participants were free to drink water during the experiment [24]. After the exercise challenge, the participants were immediately provided with RES or placebo capsules and a high glycaemic index (GI) carbohydrate meal (carbohydrate 138.03 ± 0.8 g; protein 19.4 ± 0.5 g; and fat 5.4 ± 0.1 g; 678.0 ± 18.0 kcal). The HGI diet content included corn flakes (Kellogg Co. of Great Britain Ltd., Manchester, UK), skimmed milk, white bread, jam, glucose water and water. The GI of the meal was 76.6 [25]. Muscle samples were taken immediately and 3 h after exercise to measure muscle glycogen, glucose uptake-related genes and mitochondrial biosynthetic gene expression. In addition, for a 3-h exercise recovery period, blood and gas samples were taken to analyze glucose, insulin, triglyceride, free fatty acid, glycerol concentration and respiratory exchange ratio (RER).

### 2.3. Resveratrol and Placebo Capsules

The RES used in this experiment was extracted from peanuts (160 mg/peanut) and the RES capsules were produced using self-nano-emulsifying drug delivery systems [26]. In the experiment, three RES capsules were taken each day (a total of 480 mg), which was within the safe range for the human body [27]. Each RES capsule contained a mixture of resveratrol with maltodextrin and lecithin (Taiwan Jellyfig Biotechnology Corporation, Kaohsiung, Taiwan). The content of the placebo capsules contained starch. The participants received RES supplement for 4 consecutive days (160 × 3/day, 1920 mg in total).

### 2.4. Muscle Biopsy Sampling and Glycogen Analysis

One to two mL of anesthetic (2% lignocaine) was injected for local anesthesia. Subsequently, a no. 14 muscle puncture needle (Temno, McGaw Park, IL, USA) was used to puncture the lateral femoral muscle of the participants to obtain muscle samples (approximately 20–50 mg) [28]. After the samples were taken, blood was immediately removed, and the samples were stored in liquid nitrogen. To analyze glycogen, the enzyme decomposition method was adopted [29]. First, 1 N of KOH was used to dissolve the muscle sample. Next, 0.3 M sodium acetate was used to adjust the pH level. Subsequently, amyloglucosidase (Boehringer Mannheim, Mannheim, Germany) was applied to decompose glycogen into glucose. Then, the Trider Glucose Kit (Merck, Darmstadt, Germany) was used, and a spectrophotometer (Beckman, Brea, CA, USA) OD505 nm was utilized to measure the glucosyl unit. The obtained data were depicted as standardized curves to calculate the regression equation. Finally, muscle glycogen level was obtained.

### 2.5. RNA Extraction and Real-Time RT-PCR

Real-time RT-PCR was performed to determine the levels of gene expression in the biopsied muscle tissue. Total RNA from biopsied muscle tissue was isolated using the EZNA^®^ tissue RNA kit from Omega Bio-Tek (Norcross, GA, USA) according to the manufacturer’s protocol. cDNA was synthesized from the total RNA by reverse transcription PCR using a high-capacity cDNA reverse transcription kit (Applied Biosystems, Foster City, CA, USA) according to the manufacturer’s instructions. Table 1 shows the consensus primer pairs used for β-actin, GLUT4, HKII, TBC1D1, TBC1D4, PGC-1α, SIRT1, ERR-α, NRF1, NRF2 and TFAM. Real-time RT-PCR for the determination of gene expression levels was performed using a QuantStudio™ 3 Real-Time RT-PCR System (Applied Biosystems, Foster City, CA, USA). The reaction mixture (total volume, 10 μL) contained 1×SYBR green PCR master mix, 5 μM forward primer, 5 μM reverse primer, cDNA and double deionized water, as well as commercial reagents (Applied Biosystems, Foster City, CA, USA). The thermal profile was established according to the manufacturer’s protocol: 95 °C for 10 min for enzyme activation, followed by 40 cycles of denaturing at 95 °C for 15 sand annealing and elongation at 60 °C for 1 min. The relative levels of gene expression were quantified using the 2(-delta delta CT) method, which gives a ratio of the target gene expression to the expression of stably expressed housekeeping genes [30].

### 2.6. Gas Sample Collection and Analysis

During the exercise recovery period, the participants were given a meal consisting of carbohydrates. For 3 h at every 60 min (i.e., at 60, 120 and 180 min), gas was collected by a gas analyzer (Cortex Biophysik, Nonnenstrasse, Leipzing, Germany) to analyze the amount of oxygen and carbon dioxide generated to calculate RER (RER = the amount of CO_2_ generated/VO_2_). For maximal oxygen consumption (VO_2max_) test, the participants wore masks to complete the VO_2max_ measurement on cycle ergometers (Monark, Varberg, Sweden) while using gas analyzers. The ergometers were required to maintain a rotating speed of 60 rpm. The load was initially set at 0.5 kg for 4 min and then increased by 0.5 kg at 2-min intervals until the participants were exhausted. The VO_2max_ was required to satisfy the following criteria: (1) RER > 1.10; (2) VO_2max_ variance < 2 mL/kg/min; and (3) heart rate target reaches maximum heart rate at (220-age) [31].

### 2.7. Blood Sample Collection and Analysis

The collected blood samples were centrifuged at 1000 g/10 min. The supernatant of the samples was collected and placed in a freezer at −20 °C. The blood test items consisted of glucose, insulin, non-esterified fatty acids (NEFA) and glycerol.

#### 2.7.1. Glucose Analysis Method

The glucose oxidase enzyme method was implemented. The RANDOX reagent (Laboratories Ltd., Ardmore, UK) was used. The sample was placed into a SmartSpec Plus spectrophotometer (Bio-Rad Laboratories, Hercules, CA, USA) for measurement. An automated biochemical analyzer was used for analysis at an absorbance wavelength of 340 nm and a subwavelength of 450 nm.

#### 2.7.2. Insulin Analysis Method

Insulin in the plasma was measured using a commercial reagent set (Roche Diagnostics, Mannheim, Germany). Streptavidin microparticle as well as anti-insulin AB-biotin and anti-insulin AB-Ru (bpy) 32+ were used to generate chemiluminescence. Electrochemiluminescence immunoassay (Roche Elecsys 1010/2010, Roche Diagnostics; MODULAR ANALYTICS E170, Roche Diagnostics) was adopted for analysis.

#### 2.7.3. NEFA Analysis Method

NEFA in the plasma was measured using a commercial reagent (Wako, Neuss, Germany). An automated biochemical analyzer (7020, Hitachi Science Systems, Ltd., Lbaranki, Japan) was used for testing.

#### 2.7.4. Glycerol Analysis Method

Glycerol in the plasma was measured using a commercial reagent (Randox, Co., Antrim, UK). An automated biochemical analyzer (7020, Hitachi Science Systems, Ltd., Lbaranki, Japan) was used for testing.

### 2.8. Statistics

The experimental data are presented as mean ± standard error (mean ± SE). Statistical analysis software SPSS was used for data analysis. Paired *t*-test was used to analyze the resynthesized glycogen, insulin-stimulating glucose uptake gene expression and relevant gene expression of mitochondrial biosynthesis. Blood and gas data underwent repeated measure two-way ANOVA (trial × time) to analyze changes in groups at different times. If the experimental procedure and the time point exhibited significant interaction, then simple main effects analysis was conducted. Fisher’s least significant difference was used for a post-hoc analysis. The statistical standard was set at α = 0.05.

## 3. Results

### 3.1. Resveratrol Was Unable to Alter Fat Metabolism during Cycling Exercise Recovery Period

**Figure 1:**
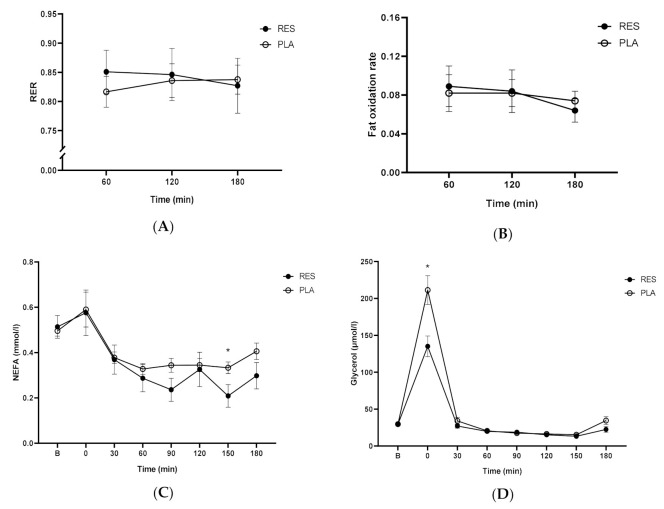
Respiratory exchange ratio (RER) (**A**), fat oxidation rate (**B**), non-esterified fatty acid (NEFA) (**C**) and glycerol (**D**) concentrations after a single bout of exercise in RES (-●-) and PLA (-○-) trials. B: immediately prior to exercise; RES: resveratrol; PLA: placebo. Values are expressed as mean ± SE, N = 9. * Significant difference against placebo (*p* < 0.05).

Figure 1 shows the changes in respiratory exchange ratio (RER) (Figure 1A), fat oxidation rate (Figure 1B), non-esterified fatty acids (NEFA) (Figure 1C) and glycerol (Figure 1D) during post-exercise recovery in both RES and placebo trials. We noticed no significant response of RER (Figure 1A, *p* > 0.05) and fat oxidation rate (Figure 1B, *p* > 0.05) during 3-h post-exercise recovery with RES supplementation. For the lipolysis data, the levels of plasma NEFA in RES trial were significantly lower than in the placebo trial at 150 min (RES 0.20 ± 0.05; Placebo 0.33 ± 0.02, *p* = 0.04) (Figure 1C). In addition, a significantly lower response of glycerol was found in RES trial immediately after exercise (RES 135.10 ± 14.04; Placebo 211.33 ± 19.56, *p* = 0.01) (Figure 1D). All plasma results indicated that short-term oral RES supplementation was unable to influence the whole-body lipolysis and gaseous exchange substrate oxidation during exercise recovery periods in young individuals.

### 3.2. Resveratrol Did Not Change Muscle Glycogen Levels Following Exercise

**Figure 2:**
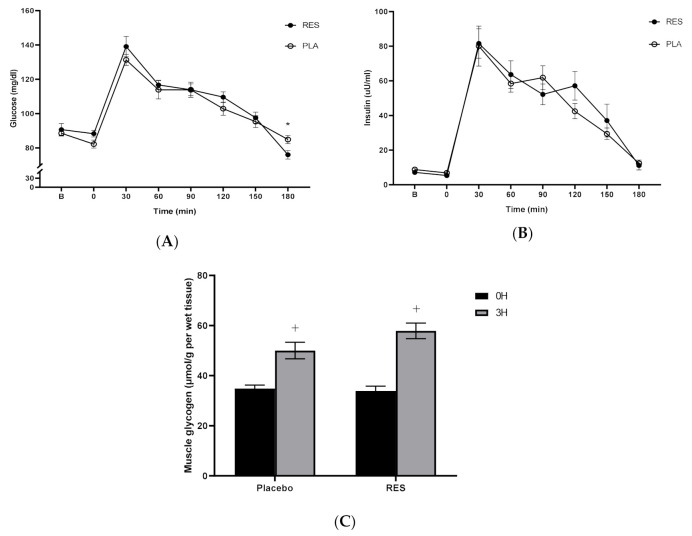
Blood glucose (**A**), insulin concentrations (**B**) and glycogen levels (**C**) after a single bout of exercise in RES and placebo trials. B: immediately prior to exercise; RES: resveratrol; PLA: placebo; 0H: immediately after exercise; 3H: 3 h after exercise. Values are expressed as mean ± SE, N = 9. * Significant difference against placebo (*p* < 0.05). + Significant difference against 0 h (immediately after exercise; *p* < 0.05).

Figure 2 displays the changes in blood glucose (Figure 2A), insulin (Figure 2B) and muscle glycogen level (Figure 2C) during the exercise recovery period immediately after a carbohydrate meal. The responses of blood glucose and insulin peaked at 30 min following exercise in both trials. Interestingly, the glucose concentration of the RES trial was significantly lower than that of the placebo at 180 min during post-exercise recovery (RES 76.00 ± 3.77, Placebo 84.89 ± 3.22, *p* = 0.002). However, we noticed that no significant response to insulin concentration was found during the 3-h post-exercise recovery period (Figure 2B, *p* > 0.05). Simultaneously, the calculated glucose area under the curve (GAUC) (4236 ± 769 mg/dL × min) and insulin area under the curve (IAUC) (6603 ± 670 μU/mL × min) values for the RES trial were not significantly different compared with the values of GAUC (3926 ± 635 mg/dl × min) and IAUC (7323 ± 969 μU/mL × min) of the placebo trial (*p* > 0.05). A significant increase in muscle glycogen level was found at 3 h compared to those at 0 h during the exercise recovery period (Placebo 3 h 50.03 ± 3.31, 0 h 34.83 ± 1.41, *p* = 0.003; RES 3 h 57.88 ± 3.14; 0 h 33.89 ± 1.94, *p* = 0.0001). However, no significant differences in glycogen level were found between resveratrol and placebo trials (Figure 2C, *p* > 0.05). Therefore, the replenishment in exercised human muscle glycogen was increased significantly after the high-intensity cycling exercise challenge in this study. However, this glycogen physiology phenomenon was not changed after resveratrol supplementation following a single bout of exercise.

### 3.3. Resveratrol Was Unable to Upregulate mRNA Expression of Glucose Utilization and Mitochondrial Biogenesis in Skeletal Muscle

**Figure 3:**
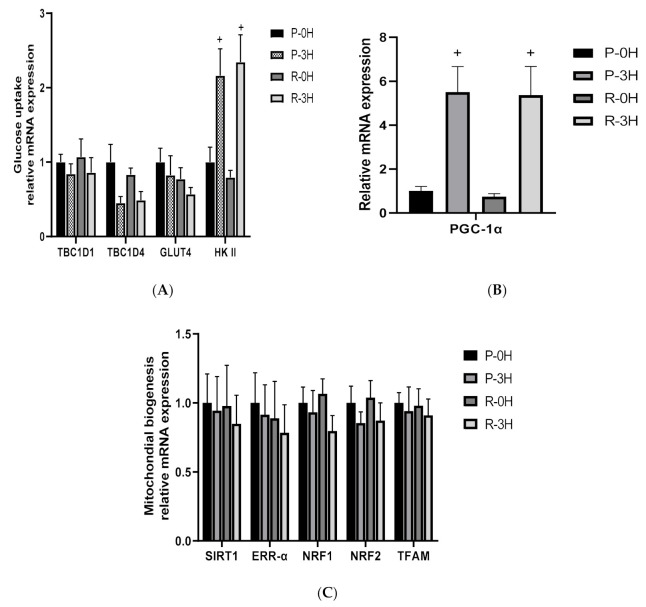
Insulin stimulates glucose uptake related gene expression (**A**), PGC-1α (**B**) and mitochondrial biogenesis-related gene expression (**C**) in vastus lateralis of human skeletal muscle after a single bout of exercise in RES and placebo trials. P-0H: immediately after exercise in placebo trial; P-3H: 3 h after exercise in placebo trial; R-0H: immediately after exercise in resveratrol trial; R-3H: 3 h after exercise in resveratrol trial. Values are expressed as mean ± SE, N = 9. + Significant difference against 0 h (immediately after exercise; *p* < 0.05).

Figure 3 shows the influence of resveratrol on the glucose uptake-related gene expression (Figure 3A), PGC1-α gene expression (Figure 3B) and expression of mitochondrial biogenesis-related genes including SIRTl, ERR-α, NRF1, NRF2 and TFAM (Figure 3C). The RT-PCR results showed that there was no difference in glucose uptake gene expression including TBC1D1, TBC1D4 and GLUT4 between RES and placebo trials immediately after exercise (0 h) and post-exercise recovery period (3 h) (Figure 3A, *p* > 0.05). However, HKII gene expression was higher at 3 h than 0 h during the exercise recovery period in both RES and placebo trials (RES 3 h 2.34 ± 0.36, 0 h 0.79 ± 0.09, *p* = 0.001; Placebo 3 h 2.16 ± 0.36, 0 h 1.0 ± 0.2, *p* = 0.009) (Figure 3A). In both trials, PGC1-α gene expressions were significantly higher at 3 h post-exercise than those immediately after exercise (0 h) (RES 3 h 5.36 ± 1.31, 0 h 0.77 ± 0.13, *p* = 0.007; Placebo 3 h 5.49 ± 1.16, 0 h 1 ± 0.20, *p* = 0.008) (Figure 3B). Nevertheless, no significant differences in gene expressions of SIRTl, ERR-α, NRF1, NRF2 and TFAM were found during the exercise recovery period between RES and placebo trials (Figure 3C, *p* > 0.05). Based on the evidence, we concluded that 4-day oral RES supplementation could not upregulate mitochondrial biogenesis gene expression of exercised human skeletal muscle in young individuals.

## 4. Discussion

Resveratrol (RES) is a natural polyphenol compound [32]. Previous cell and animal studies have shown that RES is conducive to increasing insulin sensitivity and enhancing mitochondria function [23,33]. The purpose of this human study was to demonstrate the effect of oral RES supplementation on fat oxidation, insulin sensitivity, muscle glycogen replenishment and skeletal muscle mitochondria synthesis efficiency in young adults. We reported the first human experiment on whether RES supplement affects fat and carbohydrate metabolic rates during the exercise recovery period. According to lipolysis data, it was revealed that the blood NEFA concentration was significantly reduced by RES at 150 min during the recovery period. However, no significant fat oxidation effect was observed via taking RES supplement after exercise based on the response in terms of respiratory exchange ratio (Figure 1A,B). In addition, the blood glycerol level was significantly lower in the RES trial than in the placebo trial immediately after exercise (Figure 1C). During exercise, the high demand for ATP promotes glycerol decomposition into glycolysis. We inferred that RES supplement increased the glycerol-ATP efficiency during exercise. However, during the recovery period after exercise, the RES supplement did not show the trend of increasing fat oxidation rate (Figure 1A,B). The results of fat oxidation after taking RES supplement differed from the previous study results regarding cells, mice and human experiments [34,35,36]. Randell et al. (2013) measured the effects of 7-day green tea polyphenol supplementation on fat oxidation during moderate-intensity exercise and did not discover an increased fat oxidation effect during exercise. They inferred that moderate-intensity exercise situational factors masked the physiological effects triggered by green tea polyphenols [37]. Similarly, we infer that our fat oxidation results differ from those of previous studies in which fat oxidation was promoted because the physiological effect triggered by high-intensity exercise may far outweigh that of the RES supplement. In a study by Randell et al., exercise involving a 60-min cycle, 50% W_max_, masked the effect on fat oxidation after 7-day oral green tea extract supplementation. In our study of the fat oxidation effect of 4-day RES supplementation, the results were also masked by a 60-min cycle, 70% VO_2 max_. We inferred that in this human study with a 60-min cycle, 70% VO_2max_, the fat oxidation effect should be far greater than the physiological effect triggered by a total of 1920 mg of RSE intake in 4 days; thus, no significant change in fat energy metabolism was observed during the recovery period.

Subsequently, we observed whether 4-day RES supplementation could enhance glucose utilization during the recovery period after exercise, promote whole-body insulin sensitivity or upregulate the gene expression of muscle glucose uptake involving TBC1D1, TBC1D4, GLUT4 and HKII in this study. We measured the gene expression of TBC1D1, TBC1D4 and GLUT4 immediately and 3 h after a single bout of exercise. Immediately after the session, the gene expression of TBC1D1 and TBC1D4 peaked, and they gradually declined until 3 h after exercise, but no statistical difference was observed (*p* > 0.05; Figure 3A). A previous human study showed that the protein expression of TBC1D1 and TBC1D4 significantly increased after a single bout of one-legged knee extensor exercise at 80% and 100% of workload every 5 min alternately for 60 min, whereas those of the leg without resistance exercise did not significantly change [8]. Although the present study did not collect muscle samples before exercise and therefore could not verify whether the 60-min with 70% VO_2max_ cycling exercise challenge significantly increased the gene expression of TBC1D1 and TBC1D4, we observed that during the recovery period (i.e., 0–3 h after exercise), the gene expression of TBC1D1, TBC1D4 and GLUT4 of muscle declined, showing downregulation. Kuo et al. conducted a study on mice exercising and inferred that the expression of the glucose-utilizing gene GLUT4 exhibited a trend of downregulation, possibly because it entered the protein translation cycle earlier, increasing protein translational efficiency [38]. The present study investigated whether the downregulation of TBC1D1 and TBC1D4 of human muscle is conducive to increasing the glucose utilization ability of muscle cells. We could not offer proof of protein expression because the amount of needle biopsy muscle sample from the vatus lateralis was insufficient. However, RES supplement did not alter the trends triggered by exercise. In addition, we observed that HKII gene expression significantly increased in both the RES and placebo trials (Figure 3A). HKII is a hexokinase isoform existing in muscle cells. When hexokinases are under low-oxygen or low-energy conditions, they help to convert glucose into glucose-6-phosphate to maintain glucose energy metabolism. HKII was significantly affected by the exercise intensity of 70% VO_2max_ and was significantly increased (Figure 3A). Previous studies on mice and humans have revealed a similar trend for exercise and HKII [39,40]. The current results exhibit the same trend as HKII. In other words, 70% VO_2max_ exercise substantially increased muscle glycogen concentration. However, the RES supplement did not amplify the trend triggered by exercise. However, animal experiments showed that resveratrol administration may have a protective and regulatory influence on muscle glycogen in rats (both exercising and non-exercising). The 28 rats were treated by controlling, swimming, resveratrol and resveratrol + swimming; 10 mg/kg resveratrol was given for 4 weeks and they performed a 30-min acute swimming exercise at the end of the study. All animals were decapitated, and muscle glycogen levels were measured using the immunohistochemical method [5]. In addition, the high-intensity resistance in mice with AS160/TBC1D4-Thr649Ala knock-in mutation showed that the lack of energy status after resistance exercise activated the expression of Rab GTPase-TBC1D4/AS1601 and that of the HKII gene and upregulated GLUT4 translocation to the cell membrane, increasing the utilization of musculoskeletal glucose [41]. In addition, an experiment was conducted on male Sprague-Dawley rats which were given 15 days of a high cholesterol-fructose diet and treated with 15 days of RES supplementation. The results revealed that an agonist of estradiol receptor, a critical component in RES, substantially increased after RES supplement. The experimenters stated that estradiol is the key to RES influence on upregulating GLUT4 protein expression and increasing the absorption efficiency of glucose in muscle tissue [42]. To summarize the exercise factors identified in this human study of TBC1D1, TBC1D4, GLUT4 and HK II gene expression and muscle glycogen, the outcome of this study was that the degree of influence from exercise-triggering glucose utilization gene expression was far greater than the physiological reaction from taking RES supplement.

Mitochondria, dynamic organelles that constantly fuse and divide, are a critical factor for the human body to cope with environmental stress [43]. Mitochondria synthesis involves critical transcription factors SIRT1, PGC-1α, ERR-α, NRF1 and NRF2 as well as the TFAM signaling pathway [10]. A study with cell RES intervention demonstrated that RES significantly increased the expression of mitochondrial biosynthesis regulators PGC1-α, TFAM and NRF-1, increasing the mass and the DNA content of mitochondria [44]. A C57B1/6J mice study revealed that 8 weeks of RES supplementation (400 mg/kg/day) significantly increased the activity of SIRT1 and PGC-1, enhancing mitochondrial synthesis efficiency and significantly increasing the distance run at exhaustion, suggesting increased oxidative capacity [23]. Mitochondria is a cell organelle with a high remodel rate. Adequate exercise can increase mitochondrial synthesis efficiency. However, fatigue triggered by high-intensity or overly vigorous exercise may damage mitochondria function or diminish its ability. Therefore, taking a nutritional supplement when exercising is a critical option to increase the benefit of skeletal muscle mitochondrial synthesis [45,46].

Unlike other cell and animal experimental results, in this human experiment, 4 days of RES supplementation did not upregulate the performance of mitochondrial synthesis-related gene expression in SIRT1, PGC-1α, ERR-α, NRF1, NRF2 and TFAM during the exercise recovery period (Figure 3B,C). We inferred that dosage may be a critical factor; animal and human experiments have demonstrated positive effects of using RES supplement, but with far greater dosage or longer supplementing period than the 4-consecutive-day, 1920-mg total dosage design of the present study.

In this human study, the major findings are as follows: (1) 4-day oral RES supplementation did not significantly increase the rate of whole-body fat oxidation during the exercise period. (2) The exercised muscle glycogen replenishment did not exhibit a significant increase due to RES supplement. The same trend was also observed in the whole-body insulin sensitivity of the participants or the gene expression of skeletal muscle glucose metabolism-related transcription factors TBC1D1, TBC1D4, GLUT4 or HKII. (3) RES supplement was not conducive to upregulating molecular signal SIRT1, PGC1-α, ERR-α, NRF1, NRF2 or TFAM gene expression of skeletal muscle mitochondria synthesis during the recovery period. We inferred that this was because the supplement dosage was insufficient or exercise effects masked the supplement effect. Consequently, the results demonstrated that 4 days of oral RES supplementation could not enhance exercised human muscle glycogen replenishment ability and mitochondria biosynthesis efficiency. According to previous rodent studies of the chronic/acute effects of resveratrol on ergogenic properties [5,26], further human studies will be conducted using the recommended dosage of 60 mg/d for chronic effect and 2400 mg/d for acute effect of resveratrol, which are warranted to confirm the ergogenic effects on muscle glycogen replenishment ability and mitochondria biosynthesis efficiency in exercised humans. However, this study has limitations, including the lower amount of sampling from vastus lateralis biopsies and lack of records about physical activity maintenance or exercise training diaries for all participants. In the present human study, the amount of sampling from vastus lateralis biopsies was around 20–50 mg in a human study. The mechanism variables underlying muscle physiology cannot be the same as those of animal studies. This training diary information and human muscle samples could provide additional details that could elucidate the exclusive beneficial effects of resveratrol in humans.

## Figures and Tables

**Table 1:** Primer pairs used for RT-PCR.

Gene	(Forward)	(Reverse)
*β-actin*	5′-CCTGGCACCCAGCACAAT-3′	5′-GCCGATCCACACGGAGTACT-3′
*GLUT4*	5′-CACAGTCTTCACCTTGGTCTCG-3′	5′-GTAGCTCATGGCTGGAACTCG-3′
*HKII*	5′-TTGTCCGTAACATTCTCATCGATT-3′	5′-TGTCTTGAGCCGCTCTGAGAT-3′
*TBC1D1*	5′-GTGTGGGAAAAGATGCTTAGCA-3′	5′-GTGATGACGTGGCACACCTT-3′
*TBC1D4*	5′-AGCTCCAGTGAACAGTGCAGTG-3′	5′-CACTTAGGGACTCATTGCTGC-3′
*PGC-1α*	5′-CGAGGAATATCAGCACGAGAGG-3′	5′-CATAAATCACACGGCGCTCTTC-3′
*SIRT1*	5′-TACGACGAAGACGACGACGA-3′	5′-CGCCGCCGCCGCCTCTTCC-3′
*ERR-α*	5′-TGCCAATTCAGACTCTGTGC-3′	5′-CCAGCTTCACCCCATAGAAA-3′
*NRF1*	5′-CTACTCGTGTGGGACAGCAA-3′	5′-AGCAGACTCCAGGTCTTCCA-3′
*NRF2*	5′-AAGTGACAAGATGGGCTGCT-3′	5′-TGGACCACTGTATGGGATCA-3′
*TFAM*	5′-CGCTCCCCCTTCAGTTTTGT-3′	5′-CACTCCGCCCTATAAGCATC-3′
